# Association between tirofiban administration and sex in outcomes following endovascular treatment for large vessel occlusion stroke

**DOI:** 10.3389/fneur.2026.1850978

**Published:** 2026-09-09

**Authors:** Sheng Zhou, Yongjiao Dai, Lingyu Zhang, Haoxuan Zhu, Lilan Wang, Rugang Luo, Li Yang, Li Jing, Xiao Wang, Yanying Zhang, Jiao Tu, Tao Lin, Guojian Liu, Linyu Li, Chawen Ding, Jiaxing Song, Lianjin Wang, Mingrui Zhou

**Affiliations:** 1 Department of Neurology, Renhuai People's Hospital, Zunyi, Guizhou, China; 2 Department of Neurology, Lanzhou University Second Hospital, Lanzhou, China; 3 Department of Neurology, Xinqiao Hospital and The Second Affiliated Hospital, Army Medical University (Third Military Medical University), Chongqing, China; 4 Department of Neurology, The 908th Hospital of Chinese People’s Liberation Army Joint Logistic Support Force, Nanchang, China; 5 Department of Neurology, The Second Affiliated Hospital of Chongqing Medical University, Chongqing, China; 6 The First Clinical Medical School, Hubei University of Chinese Medicine, Wuhan, China

**Keywords:** acute ischemic stroke, clinical outcomes, endovascular treatment, large vessel occlusion, sex differences, tirofiban

## Abstract

**Background:**

Intravenous tirofiban administered before endovascular treatment (EVT) did not improve 90-day outcomes compared to placebo in the RESCUE BT trial, and sex-specific heterogeneity remained uncertain. In this study, we aimed to evaluate the association between sex and tirofiban administration before EVT among patients enrolled in the RESCUE BT trial.

**Materials and methods:**

This *post hoc* analysis used data from the RESCUE BT trial, which enrolled 948 patients with large vessel occlusion (LVO). Patients received intravenous tirofiban or a placebo before EVT. Comparisons between different groups were conducted using logistic regression. Additionally, the interaction between sex and treatment was tested. The primary effectiveness outcome was a modified Rankin Scale (mRS) score of 0–2 at 90 days; safety outcomes included symptomatic intracranial hemorrhage (sICH) within 48 h and mortality at 90 days.

**Results:**

Of the 948 patients, 391 (41.2%) were women. The interaction between sex and treatment for a 90-day mRS score of 0–2 was not statistically significant (*P* for interaction = 0.103). However, in the tirofiban arm, women were less likely to achieve an mRS score of 0–2 at 90 days (adjusted odds ratio (aOR), 0.57; 95% confidence interval (CI), 0.36–0.90; *p* = 0.02) compared to men. In analyses stratified by sex, tirofiban was not associated with improved functional outcomes compared to placebo in either women or men. No sex-related differences were observed in symptomatic intracranial hemorrhage within 48 h or mortality at 90 days.

**Conclusion:**

In this exploratory analysis, no significant interaction between sex and treatment was identified, indicating that the association between tirofiban and 90-day outcomes did not differ by sex. Although women in the tirofiban arm had lower odds of achieving an mRS score of 0–2 at 90 days than men, this finding should be interpreted as hypothesis-generating and warrants further validation.

## Introduction

Several studies have suggested that sex is associated with both pathophysiological and sociobiological differences among patients with acute ischemic stroke (AIS) (1–3), which may contribute to a higher proportion of women experiencing worse prognosis (4, 5). Given that women live longer than men, they are older at the time of AIS onset (6). Furthermore, misdiagnosis, delays in stroke center arrival, and reduced social support may contribute to the relatively worse outcomes observed among women (7–9). However, this potential sex-based difference has been reported inconsistently among AIS patients undergoing endovascular thrombectomy (EVT). While some researchers have found no difference in outcomes between men and women with anterior large vessel occlusion (LVO) after EVT (10–12), others such as de Ridder et al. have suggested that women in the MR CLEAN trial appeared to have a slightly more unfavorable profile (13).

Tirofiban is a fast-acting, highly selective, low-molecular-weight, non-peptide glycoprotein IIb/IIIa receptor inhibitor (14). Several clinical trials conducted in recent years have evaluated the effectiveness of tirofiban, either alone or as an adjunct to intravenous thrombolysis and/or EVT, in patients with AIS (15–18). However, our previous study, the RESCUE BT trial, did not show a significant improvement in 90-day clinical outcomes among patients undergoing EVT after receiving intravenous tirofiban compared to those who received a placebo (19). In addition, the subgroup analysis of this trial showed no notable differences in outcomes between male and female individuals. Nevertheless, no further investigation of sex-based differences was conducted, particularly in the tirofiban arm. To the best of our knowledge, no prior studies have evaluated the association between tirofiban treatment and sex among LVO patients undergoing EVT.

Gender equality is receiving increased emphasis recent times, highlighting the importance of exploring the disparities between sexes. Therefore, in this study, we aimed to investigate the association between tirofiban treatment and sex among patients enrolled in the RESCUE BT trial.

## Materials and methods

### Study design and patient selection

This study was a *post hoc* analysis of the RESCUE BT trial, which was an investigator-initiated, multicenter, randomized, double-blind, placebo-controlled trial that enrolled 948 patients with stroke and proximal intracranial large vessel occlusion who presented within 24 h of the time they were last known to be well. The trial was conducted across 55 comprehensive stroke centers in China. Recruitment took place from 10 October 2018 through 31 October 2021, and the final follow-up was completed on 15 January 2022. Patients enrolled in the trial received intravenous tirofiban or a placebo before undergoing EVT. A more detailed description of the trial has been provided in a previous publication (19). All patients or their legal representatives provided written informed consent before enrollment. The initial study was carried out in accordance with the Declaration of Helsinki and was approved by the ethics committees of all participating centers.

The inclusion criteria were as follows (1): a National Institutes of Health Stroke Scale (NIHSS) score of 30 or less (range, 0–42; higher scores indicate more severe neurologic deficits) (2); an Alberta Stroke Program Early CT Score (ASPECTS) of 6 or more (range, 0–10; higher scores suggest a smaller infarct core); and (3) occlusion of the intracranial internal carotid artery or the first or second segment of the middle cerebral artery confirmed by computed tomography angiography (CTA), magnetic resonance angiography (MRA), or digital subtraction angiography. The primary exclusion criteria were dual antiplatelet therapy within 1 week of the index stroke or receipt of intravenous thrombolysis after stroke onset.

### Data collection

Patients were stratified by self-reported sex (or the sex reported by a surrogate) using data from the original study. Baseline characteristics included demographics, medical history, clinical examination data, stroke characteristics, stroke etiology, occlusion site, the American Society of Interventional and Therapeutic Neuroradiology/Society of Interventional Radiology (ASITN/SIR) grade, and time metrics in minutes (from the last known well to puncture and from puncture to recanalization or procedure completion). Medical history included hypertension, diabetes, hyperlipidemia, previous stroke, atrial fibrillation, and current or previous smoking status. Clinical examination data included systolic blood pressure, diastolic blood pressure, and glucose levels at admission. Stroke characteristics included baseline National Institutes of Health Stroke Scale (NIHSS) scores and the Alberta Stroke Program Early Computed Tomography Score (ASPECTS). Stroke etiology was classified in accordance with the Trial of ORG 10172 in Acute Stroke Treatment (TOAST) classification.

### Outcome measures

The primary effectiveness outcome was functional independence, defined as a modified Rankin Scale (mRS) score of 0–2 at 90 days. The secondary effectiveness outcomes included the distribution of mRS scores at 90 days, an mRS score of 0–1 at 90 days (excellent outcome), and an mRS score of 0–3 at 90 days (favorable functional outcome). Safety outcomes included the incidence of symptomatic intracranial hemorrhage (sICH), which was assessed according to the Heidelberg Bleeding Classification within 48 h (20). Other safety variables were any intracranial hemorrhage (aICH) and mortality within 90 days.

### Statistical analysis

Patients were categorized by self-reported sex (or the sex reported by a surrogate) as male or female. Baseline characteristics were compared overall, within the sex strata, and within the therapy strata. Supplementary Figures 1, 2 illustrate the patient division. The normality of distribution for continuous variables was assessed using the Kolmogorov–Smirnov test. They were presented as medians with interquartile ranges (IQRs) and compared across groups using the Kruskal–Wallis H test. Categorical variables were presented as frequencies and percentages (%) and compared using the chi-squared or Fisher’s exact test.

We used the logistic regression model to estimate odds ratios (ORs) with 95% confidence intervals (CIs) for the primary outcome. Specifically, binary logistic regression was used for dichotomous 90-day outcomes, including mRS scores of 0–2, 0–3, and 0–1, mortality, sICH, and any intracranial hemorrhage (aICH). The association with the distribution of 90-day mRS scores was assessed using ordinal logistic regression. To control for potential confounding, adjusted logistic regression models were adjusted for confounders, including atrial fibrillation, baseline NIHSS score, baseline ASPECTS, time from the last known well to puncture, occlusion site, smoking status, and stroke etiology. To assess whether sex modified the association between treatment assignment and clinical outcomes, an interaction term between sex and randomized treatment (sex*treatment arm) was included in the regression models. As a sensitivity analysis to address potential residual confounding by age between women and men, the multivariable regression models were repeated with age included as an additional covariate. The remaining adjustment variables were identical to those in the primary models. The *p*-values for interaction were obtained from the sex-by-treatment interaction term. Furthermore, the same pattern of analysis was conducted to investigate sex-based differences among large artery atherosclerosis (LAA) patients in our study.

Exploratory subgroup analyses were performed in the tirofiban arm based on age (≤67 vs. >67 years), baseline NIHSS score (≤16 vs. > 16), baseline ASPECTS (≤7 vs. > 7), stroke etiology (LAA, CE, or others), occlusion site (intracranial ICA, MCA-M1, or MCA-M2), and onset-to-randomization time (<400 vs. ≥400 min). Adjusted odds ratios and 95% CIs for achieving an mRS score of 0–2 at 90 days were estimated using multivariable logistic regression models adjusted for the same covariates as those used in the multivariable analysis, including atrial fibrillation, baseline NIHSS score, baseline ASPECTS, time from the last known well to puncture, occlusion site, smoking status, and stroke etiology. Heterogeneity across subgroups was assessed using the corresponding interaction terms.

Given the exploratory and *post hoc* nature of this study and the absence of a prospectively defined family of hypotheses for the multiple secondary, subgroup, interaction, and marginal effect analyses, no formal adjustment for multiple comparisons was made. The principal interpretation of the results was therefore restricted to the analyses of a 90-day mRS score of 0–2 and 90-day mortality. Analyses of all other outcomes, as well as subgroup, interaction, and marginal effect analyses, were considered exploratory and hypothesis-generating. The corresponding *p*-values should not be interpreted as confirmatory evidence.

Two-sided p-values less than 0.05 and 95% confidence intervals (95% CIs) for odds ratios (ORs) that excluded 1.0 were considered statistically significant. All statistical analyses were performed using SPSS (IBM SPSS Statistics for Windows, version 26.0) and R (R Project for Statistical Computing, version 4.4.2).

## Results

### Patient characteristics

The baseline characteristics of the enrolled patients stratified by sex are shown in Table 1. Of the 948 patients enrolled in the trial, 391 (41.2%) were women. It could be observed that, among all patients, female patients were older, had a higher prevalence of atrial fibrillation, had a higher incidence of cardioembolism (CE), had higher levels of glucose at admission, and exhibited more severe stroke syndromes (baseline NIHSS score 17 vs. 15) compared to male patients. In contrast, female patients were less likely to have a smoking history and had shorter procedure times, including shorter times from last known well to puncture and from puncture to recanalization or procedure completion. All the differences between female and male patients described above were statistically significant (*p* < 0.05). Other details are shown in Table 1, as no significant differences in the remaining characteristics were observed between the sexes. LAA patients had the same sex-related differences in baseline characteristics as the overall cohort, except for the time from puncture to recanalization or procedure completion. In addition, among LAA patients, women had a higher prevalence of diabetes and higher serum glucose levels. Other baseline characteristics of LAA patients are shown in Supplementary Table 1.

**Table 1:** Baseline characteristics between male and female patients.

Baseline characteristics	Sex difference		*p*-value
	Female patients (*n* = 391)	Male patients (*n* = 557)	
Age, median (IQR)	71 (63–77)	64 (55–72)	<0.001
Hypertension, *n* (%)	221 (56.5)	303 (54.4)	0.517
Diabetes, *n* (%)	98 (25.1)	106 (19.0)	0.026
Hyperlipidemia, *n* (%)	57 (14.6)	75 (13.5)	0.626
Previous stroke, *n* (%)	69 (17.6)	92 (16.5)	0.648
Atrial fibrillation, *n* (%)	182 (46.5)	131 (23.5)	<0.001
Smoking, *n* (%)	2 (0.5)	220 (39.5)	<0.001
SBP, median (IQR), mmHg	144 (126–161)	146 (130–160)	0.285
DBP, median (IQR), mmHg	82 (73–93)	85 (76–95)	0.019
Glucose, median (IQR), mmol/l	7.2 (6.1–9.1)	6.7 (5.6–8.3)	<0.001
Prestroke mRS, *n* (%)			0.644
0–1	380 (97.2)	544 (97.7)	
≥ 2	11 (2.8)	13 (2.3)	
Stroke characteristics			
Baseline NIHSS, median (IQR)	17 (13–20)	15 (10–19)	<0.001
Baseline ASPECTS, median (IQR)	8 (7–9)	8 (7–9)	0.589
Stroke etiology, *n* (%)			<0.001
Large artery atherosclerosis	124 (31.7)	311 (55.8)	
Cardioembolism	227 (58.1)	179 (32.1)	
Other or Unknown	40 (10.2)	67 (12.0)	
Occlusion site, *n* (%)			0.097
ICA	93 (23.8)	101 (18.1)	
MCA-M1	241 (61.6)	374 (67.1)	
MCA-M2	57 (14.6)	82 (14.7)	
Time metrics in minutes, median (IQR), min			
Last known well to puncture	350 (237–565)	439 (274–673)	<0.001
Puncture to recanalization or procedure completion	63 (39–98)	71 (43–115.5)	0.007

Data are presented as medians (interquartile range) for continuous variables and n (%) for categorical variables.

ASPECTS, Alberta Stroke Program Early Computed Tomography Score; CE, cardioembolism; DBP, diastolic blood pressure; ICA, internal carotid artery; IQR, interquartile range; LAA, large artery atherosclerosis; MCA, middle cerebral artery; mRS, modified Rankin Scale; NIHSS, National Institutes of Health Stroke Scale; SBP, systolic blood pressure.

### Female versus male patients, stratified by treatment arm

There were 463 patients randomized to the tirofiban arm (43.2% were women and 56.8% were men), with the remaining 485 patients randomized to the placebo arm (Table 2). Several differences in baseline characteristics were observed between male and female patients in both treatment arms. Compared to male patients, female patients were older, less likely to have a smoking history and had a shorter procedure time, but they had a significantly higher prevalence of atrial fibrillation and CE stroke etiology. Similar differences were observed among LAA patients (Supplementary Table 2). These differences are consistent with those shown in Table 1.

**Table 2:** Baseline characteristics by sex, stratified by treatment arm.

Baseline characteristics	Tirofiban (*n* = 463)			Placebo (*n* = 485)		
	Female (*n* = 200)	Male (*n* = 263)	*P*-value	Female (*n* = 191)	Male (*n* = 294)	*P*-value
Age, median (IQR)	72 (62–77)	65 (55–72)	<0.001	71 (64–77)	64 (55–72)	<0.001
Hypertension, *n* (%)	111 (55.5)	140 (53.2)	0.63	110 (57.6)	163 (55.4)	0.64
Diabetes, *n* (%)	55 (27.5)	44 (16.7)	0.005	43 (22.5)	62 (21.1)	0.71
Hyperlipidemia, *n* (%)	34 (17.0)	40 (15.2)	0.6	23 (12.0)	35 (11.9)	0.96
Previous stroke, *n* (%)	34 (17.0)	38 (14.4)	0.45	35 (18.3)	54 (18.4)	0.99
Atrial fibrillation, *n* (%)	101 (50.5)	65 (24.7)	<0.001	81 (42.4)	66 (22.4)	<0.001
Smoking, *n* (%)	0 (0)	100 (38.0)	<0.001	2 (1.0)	120 (40.8)	<0.001
SBP, median (IQR), mmHg	145 (126–164)	145 (130–160)	0.96	141 (126–158)	146 (131–160)	0.11
DBP, median (IQR), mmHg	83 (75–94)	84 (76–93)	0.55	81 (73–92)	85 (77–98)	0.009
Glucose, median (IQR), mmol/l	7.4 (6.2–9.7)	6.7 (5.4–7.8)	<0.001	7.1 (5.8–8.8)	6.8 (5.7–8.7)	0.258
Prestroke mRS, n (%)						
0–1	196 (98.0)	255 (97.0)	0.49	184 (96.3)	289 (98.3)	0.17
≥2	4 (2.0)	8 (3.0)		7 (3.7)	5 (1.7)	
Stroke characteristics						
Baseline NIHSS, median (IQR)	16 (12–20)	15 (11–18)	0.002	17 (14–20)	15 (10–19)	<0.001
Baseline ASPECTS, median (IQR)	8 (7–9)	8 (7–9)	0.42	7 (6–9)	8 (7–9)	0.12
Stroke etiology, n (%)			<0.001			<0.001
Large artery atherosclerosis	64 (32.0)	133 (50.6)		60 (31.4)	178 (60.5)	
Cardioembolism	119 (59.5)	93 (35.4)		108 (56.5)	86 (29.3)	
Other or unknown	17 (8.5)	37 (14.1)		23 (12.0)	30 (10.2)	
Occlusion site, n (%)			0.43			0.16
ICA	47 (23.5)	49 (18.6)		46 (24.1)	52 (17.7)	
MCA-M1	128 (64.0)	177 (67.3)		113 (59.2)	197 (67.0)	
MCA-M2	25 (12.5)	37 (14.1)		32 (16.8)	45 (15.3)	
Time metrics in minutes, median (IQR), min						
Last known well to puncture	366 (263–568)	435 (280–680)	0.046	325 (205–565)	440 (270–662)	<0.001
Puncture to recanalization or procedure completion	65 (39–98)	68 (42–108)	0.27	60 (39–100)	75 (45–121)	0.009

Data are presented as medians (interquartile range) for continuous variables and n (%) for categorical variables.

ASPECTS, Alberta Stroke Program Early Computed Tomography Score; DBP, diastolic blood pressure; ICA, internal carotid artery; IQR, interquartile range; MCA, middle cerebral artery; mRS, modified Rankin Scale; NIHSS, National Institutes of Health Stroke Scale; SBP, systolic blood pressure.

**Figure 1:**
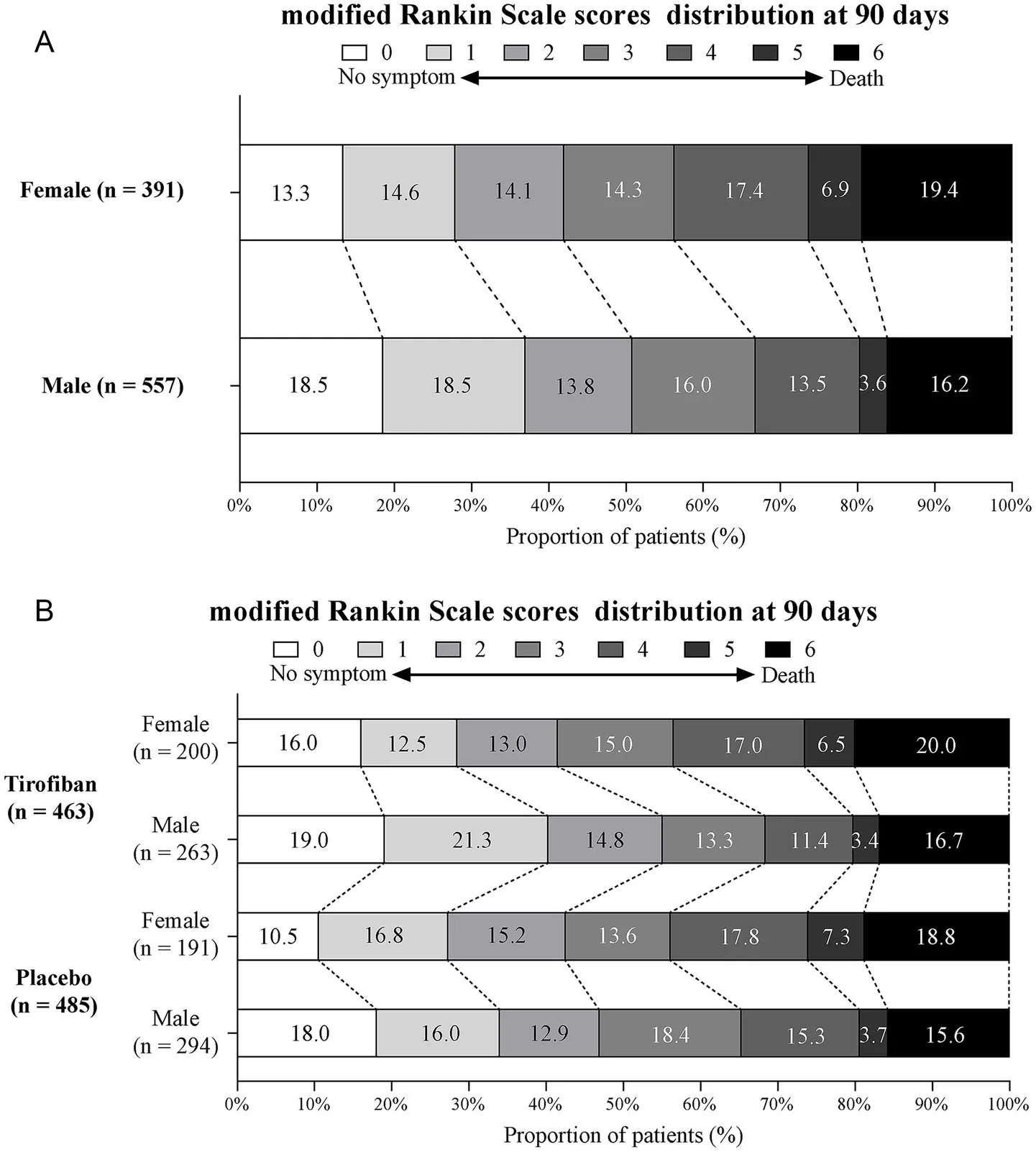
Distribution of modified Rankin Scale scores at 90 days. **(A)** Distribution of modified Rankin Scale scores at 90 days, stratified by sex. The distribution of modified Rankin Scale scores at 90 days among male and female patients. Clinical outcomes at the 90-day follow-up are shown for male and female patients. **(B)** Distribution of modified Rankin Scale scores at 90 days by sex, stratified by treatment arm. Scores on the modified Rankin Scale range from 0 to 6, with 0 indicating no symptoms; 1, symptoms without clinical disability; 2, slight disability; 3, moderate disability; 4, moderately severe disability; 5, severe disability; and 6, death.

Clinical outcomes are presented in Table 3. The interaction test showed no evidence that sex modified the association between tirofiban treatment and a 90-day mRS score of 0–2 (*P* for interaction = 0.103). Among patients assigned to the tirofiban arm, women had lower adjusted odds of achieving an mRS score of 0–2 at 90 days than men (aOR, 0.57; 95% CI, 0.36–0.90; *p* = 0.02). In a sensitivity analysis with additional adjustment for age, the results were generally consistent. The interaction remained nonsignificant (*P* for interaction = 0.083; Supplementary Table 3). Women in the tirofiban arm remained less likely than men to achieve an mRS score of 0–2 at 90 days (*p* = 0.045), whereas no corresponding association was observed in the placebo arm (*p* = 0.777). No significant sex-related difference was observed in 90-day mortality (aOR, 0.91; 95% CI, 0.51–1.61; *p* = 0.74), and the interaction was also not statistically significant (*P* for interaction = 0.811). In the tirofiban arm, stroke etiology did not significantly modify the association between sex and the likelihood of achieving an mRS score of 0–2 at 90 days (*P* for interaction = 0.237, Supplementary Table 4). In addition, the distribution of mRS scores at 90 days is illustrated in Figure 1B. Exploratory analyses of the ordinal mRS distribution, mRS scores of 0–1 and 0–3, sICH, and any intracranial hemorrhage did not demonstrate a consistent pattern of sex-related differences. Although women had lower adjusted odds of achieving an mRS score of 0–1 in the tirofiban arm (aOR, 0.55; 95% CI, 0.34–0.89; *p* = 0.01), the unadjusted difference in the ordinal mRS distribution was attenuated after multivariable adjustment (Figure 1B and Table 3). No statistically significant sex-by-treatment interactions were identified for any secondary effectiveness or safety outcomes (all *P* for interaction >0.05; Table 3). In the placebo arm, sex was not associated with a 90-day mRS score of 0–2 or 90-day mortality after adjustment. The remaining exploratory outcomes also showed no consistent evidence of sex-related differences. In the sensitivity analysis restricted to patients with LAA, no consistent sex-related differences were observed in either treatment arm (Supplementary Table 5 and Supplementary Figures 3, 4). Analogously, the sex-by-treatment interaction was not statistically significant in the LAA subgroup.

**Table 3:** Outcomes by sex, stratified by treatment arm.

Outcomes	Tirofiban (*n* = 463)						Placebo (*n* = 485)						*P* for interaction^d^
	Female (*n* = 200)	Male (*n* = 263)	OR (95% CI)	*P-*value	aOR (95% CI)^a^	*P*-value	Female (*n* = 191)	Male (*n* = 294)	OR (95% CI)	*P*-value	aOR (95% CI) ^a^	*P*-value	
Primary effectiveness outcome													
An mRS score of 0–2 at 90 days, n (%)^b^	83 (41.5)	145 (55.1)	0.58 (0.40–0.84)	0.004	0.57 (0.36–0.90)	0.02	81 (42.4)	138 (46.9)	0.83 (0.58–1.20)	0.33	0.95 (0.60–1.49)	0.82	0.103
Secondary effectiveness outcomes													
mRS scores at 90 days, median (IQR) ^c^	3 (1–5)	2 (1–4)	0.65 (0.47–0.90)	0.009	0.72 (0.49–1.05)	0.09	3 (1–5)	3 (1–4)	0.71 (0.52–0.98)	0.04	0.92 (0.64–1.34)	0.69	0.465
An mRS score of 0–1 at 90 days, n (%)^b^	58 (29.0)	110 (41.8)	0.57 (0.38–0.84)	0.005	0.55 (0.34–0.89)	0.01	55 (28.8)	102 (34.7)	0.76 (0.51–1.13)	0.18	0.85 (0.52–1.38)	0.50	0.219
An mRS score of 0–3 at 90 days, *n* (%)^b^	113 (56.5)	180 (68.4)	0.60 (0.41–0.88)	0.008	0.67 (0.42–1.06)	0.09	107 (56.0)	192 (65.3)	0.68 (0.47–0.98)	0.04	0.89 (0.57–1.40)	0.62	0.403
Safety outcomes													
Mortality at 90 days, n (%)^b^	40 (20.0)	44 (16.7)	1.24 (0.77–2.00)	0.37	0.91 (0.51–1.61)	0.74	36 (18.8)	46 (15.6)	1.25 (0.77–2.02)	0.36	0.90 (0.52–1.56)	0.71	0.811
sICH within 48 h, n (%)^b^	20 (10.0)	25 (9.5)	1.05 (0.57–1.96)	0.87	0.78 (0.38–1.59)	0.49	13 (6.8)	18 (6.1)	1.12 (0.54–2.35)	0.76	0.80 (0.34–1.91)	0.62	0.923
Any ICH, n (%)^b^	78 (39.0)	83 (31.7)	1.38 (0.94–2.03)	0.10	1.08 (0.69–1.69)	0.75	60 (31.6)	75 (25.6)	1.34 (0.90–2.01)	0.15	1.00 (0.62–1.60)	0.99	0.696

Data are presented as *n* (%) or medians (interquartile range), as appropriate.

a Adjusted for atrial fibrillation, baseline NIHSS score, baseline ASPECTS, time from last known well to puncture, occlusion site, smoking status, and stroke etiology.

b Odds ratios were estimated using binary logistic regression models.

c The common odds ratio was estimated using ordinal logistic regression models and indicates the odds of improvement toward a lower mRS score. Men served as the reference group; therefore, a common odds ratio greater than 1 indicates more favorable functional outcomes in women.

d *p*-values for interaction were derived from the sex-by-treatment interaction term in the corresponding adjusted regression models.

ASPECTS, Alberta Stroke Program Early Computed Tomography Score; CI, confidence interval; ICH, intracranial hemorrhage; IQR, interquartile range; mRS, modified Rankin Scale; NIHSS, National Institutes of Health Stroke Scale; OR, odds ratio; sICH, symptomatic intracranial hemorrhage.

### Tirofiban versus placebo, stratified by sex

Among the 391 female patients, 200 received tirofiban and 191 received placebo. Baseline characteristics were generally balanced between the treatment groups, as were those among male patients (Supplementary Table 6), consistent with the randomization in the original trial.

**Figure 2:**
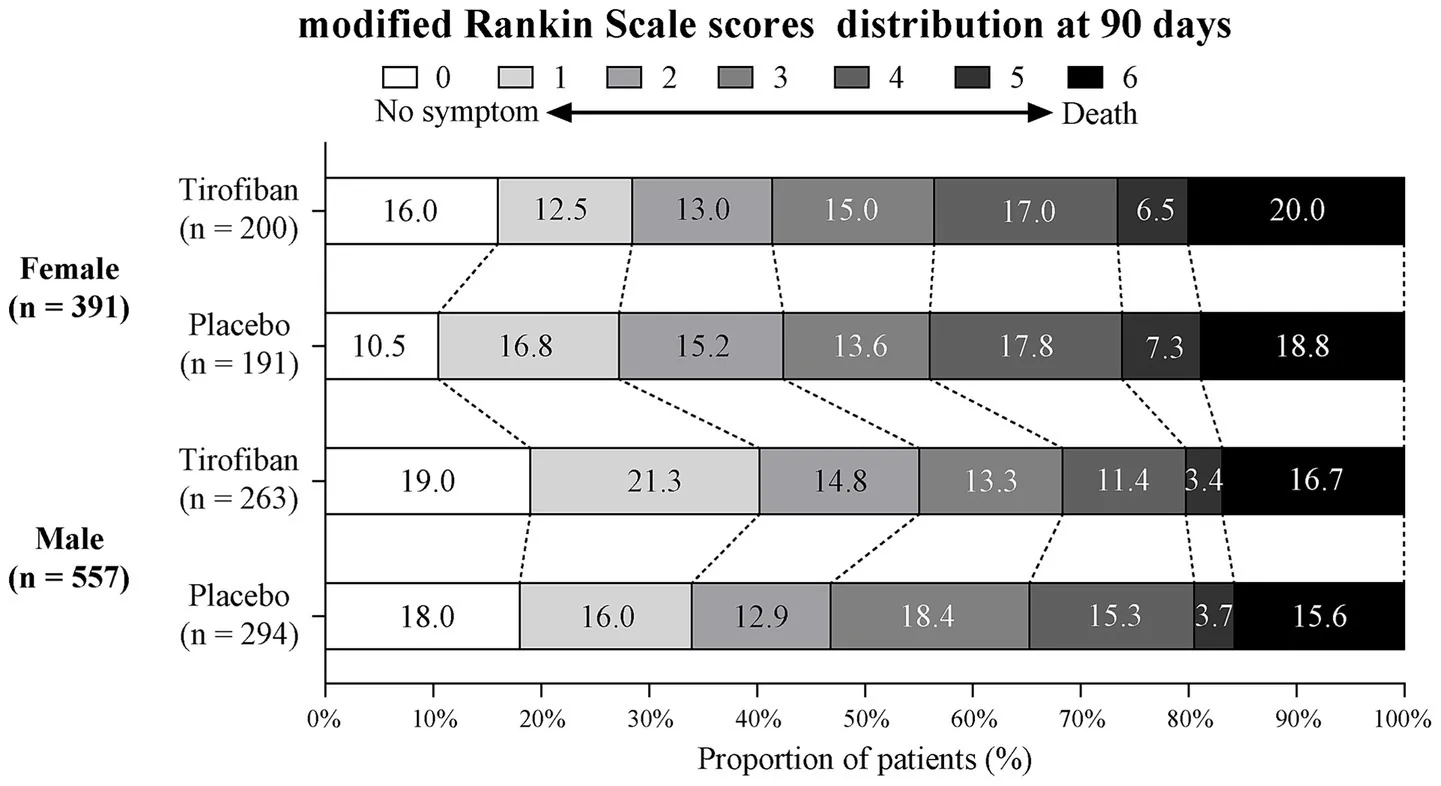
Distribution of modified Rankin Scale scores at 90 days by treatment arm, stratified by sex. Scores on the modified Rankin Scale range from 0 to 6, with 0 indicating no symptoms; 1, symptoms without clinical disability; 2, slight disability; 3, moderate disability; 4, moderately severe disability; 5, severe disability; and 6, death.

Among women, tirofiban was not associated with an mRS score of 0–2 at 90 days compared to placebo (*p* = 0.70), and no difference was observed in 90-day mortality (*p* = 0.68; Supplementary Table 7). Among men, the adjusted estimate for a 90-day mRS score of 0–2 favored tirofiban but remained imprecise, with the confidence interval including the null value (*p* = 0.06). No association with 90-day mortality was observed (*p* = 0.84). Analyses of the ordinal mRS distribution, mRS scores of 0–1 and 0–3, sICH, and any intracranial hemorrhage were exploratory and did not show consistent treatment-related differences within either sex stratum. The distribution of mRS scores at 90 days is shown in Figures 1A, 2. Figure 1A illustrates all patients stratified by sex, whereas Figure 2 illustrates patients by treatment arm, stratified by sex. Consistent with the interaction analysis reported above, there was no evidence that the association between tirofiban and clinical outcomes differed by sex (Table 3).

### Exploratory subgroup and marginal effect analyses

**Figure 3:**
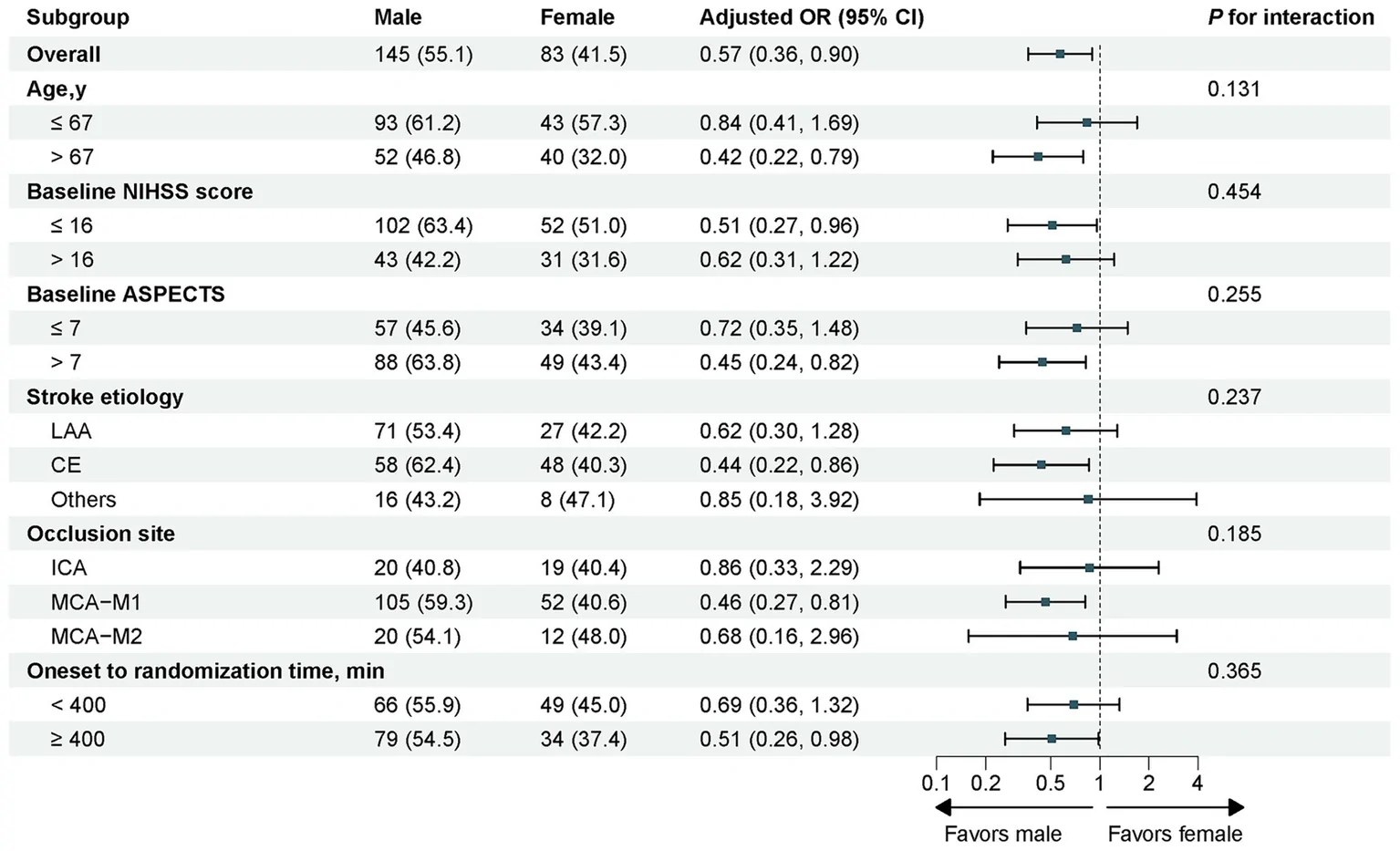
Subgroup analysis. The forest plot presents adjusted *ORs* and 95% *CIs* for achieving an mRS score of 0–2 at 90 days among women compared to men in the tirofiban arm. The numbers and percentages represent the patients who achieved an mRS score of 0–2 within each sex and subgroup. Adjusted *ORs* were estimated using multivariable logistic regression models adjusted for atrial fibrillation, baseline NIHSS score, baseline ASPECTS, the time from last known well to puncture, occlusion site, smoking status, and stroke etiology. Squares indicate point estimates, horizontal lines indicate 95% *CIs*, and the vertical line indicates an *OR* of 1. *p*-values for interaction were derived from the interaction term between sex and the corresponding subgroup variable. Abbreviations: ASPECTS, Alberta Stroke Program Early Computed Tomography Score; CE, cardioembolism; *CI*, confidence interval; ICA, internal carotid artery; LAA, large artery atherosclerosis; MCA, middle cerebral artery; mRS, modified Rankin Scale; NIHSS, National Institutes of Health Stroke Scale; *OR*, odds ratio.

Exploratory subgroup analyses were conducted in the tirofiban arm to assess whether the association between sex and a 90-day mRS score of 0–2 varied across different clinical characteristics (Figure 3). No statistically significant interaction was observed across subgroups defined by age, baseline NIHSS score, baseline ASPECTS, stroke etiology, occlusion site, or onset-to-randomization time (all *P* for interaction >0.05).

The exploratory marginal effect analyses showed that the predicted probability of achieving an mRS score of 0–2 generally decreased with increasing age, baseline NIHSS score, and puncture-to-reperfusion time, as well as with decreasing baseline ASPECTS, in both treatment arms (Supplementary Figures 5, 7). Corresponding inverse patterns were observed for predicted 90-day mortality (Supplementary Figures 6, 8). However, an isolated interaction between sex and baseline ASPECTS was observed for 90-day mortality in the tirofiban arm (*P* for interaction = 0.020; Supplementary Figure 6B). This association was not replicated in the placebo arm or consistently observed for other outcomes. No other consistent interaction was identified.

## Discussion

In this *post hoc* analysis of the RESCUE BT trial, no significant interaction between sex and tirofiban administration was identified for a 90-day mRS score of 0–2 or mortality, providing no evidence that the association between tirofiban and outcomes differed between women and men. An exploratory analysis showed that women assigned to tirofiban had lower odds of achieving a 90-day mRS score of 0–2 than men. However, given the exploratory and post hoc nature of these analyses, the findings should be interpreted cautiously and regarded as hypothesis-generating.

Although sex-based differences have been extensively examined, it remains unclear whether sex modifies clinical outcomes among patients with ischemic stroke, including those undergoing EVT. Previous studies have provided conflicting results. Subanalyses of MR CLEAN (The Multicenter Randomized Clinical Trial of Endovascular Treatment for Acute Ischemic Stroke in the Netherlands) and the DEFUSE 3 trial (The Endovascular Therapy Following Imaging Evaluation for Ischemic Stroke) found heterogeneity in treatment effects by sex, with women experiencing worse clinical outcomes (13, 21). In contrast, other analyses found no significant association between sex and clinical outcomes among patients with ischemic stroke (10, 11, 22). In our analysis, the sex-related difference in the tirofiban arm was mainly observed for a 90-day mRS score of 0–2 and was not consistently evident across the mRS distribution or safety outcomes. Moreover, interaction analysis did not indicate that sex modified the association between tirofiban and clinical outcomes. Therefore, the present findings do not establish a definitive sex-specific response to tirofiban treatment but identify a possible signal that warrants further investigation. Several previous studies have suggested that social factors, including misdiagnosis, delays in medical procedures, and a lower likelihood of transfer to intensive care units, may contribute to worse clinical outcomes among women (7, 8, 23). However, the data we collected did not reflect these discrepancies between sex. In addition, the EVT procedure time was significantly shorter among women in both treatment arms.

In our study, women were more likely to have atrial fibrillation and cardioembolic stroke, whereas LAA stroke was more prevalent among men, which is consistent with previous studies (12, 24). Previous evidence suggests that the benefit associated with tirofiban may be attenuated in patients with cardioembolic stroke (25). Although differences in thrombus composition across stroke etiologies may provide a plausible explanation for the observed findings (26, 27), the interaction analysis showed no clear evidence that stroke etiology modified the association between sex and functional independence in the tirofiban arm. Moreover, thrombus histology and platelet composition were not assessed in the RESCUE BT trial. Thus, the present findings do not provide sufficient support for stroke etiology or thrombus composition as explanations for the observed sex-related difference, which remain speculative and require further investigation.

In addition, 41.24% of patients enrolled in the RESCUE BT trial were women. The relatively lower proportion of women enrolled in stroke clinical trials has been consistently observed (10–12). Furthermore, systematic reviews have suggested that women account for only approximately 38–40% of participants in stroke RCTs and are underrepresented by approximately 5 percentage points relative to their expected proportion in the underlying population (28, 29). This underrepresentation limits the precision of sex-specific estimates of treatment effects and may contribute to the persistent uncertainty regarding whether and how sex modifies the impact of acute stroke therapies.

Regarding safety outcomes, no sex-related differences were observed in 90-day mortality, sICH within 48 h, or any intracranial hemorrhage. Among the exploratory marginal effect analyses, an isolated significant interaction between sex and baseline ASPECTS was observed for 90-day mortality in the tirofiban arm. However, no consistent pattern of interaction was identified across treatment arms or outcomes. Given the exploratory nature of these analyses, the multiple interaction tests performed, and the absence of adjustment for multiple comparisons, this isolated finding may have occurred by chance and should not be overinterpreted.

## Limitations

Our study has several limitations. First, as a *post hoc* analysis, our study remains vulnerable to unmeasured confounding despite comprehensive statistical adjustments for a wide range of known prognostic factors. The statistical power of the sex-specific analyses was limited, particularly in the LAA subgroup, in which the number of men was substantially greater than that of women. Therefore, this limited statistical power may reduce the robustness of our findings. Second, multiple outcomes, subgroup comparisons, and interaction analyses were evaluated without formal adjustment for multiple comparisons. Although these analyses were undertaken to explore potentially relevant clinical characteristics, the possibility of chance findings cannot be excluded. Accordingly, results beyond the primary functional outcome and safety outcome should be interpreted as exploratory and require independent confirmation. Third, data on sociobehavioral, socioeconomic, and psychological factors, which may influence clinical outcomes differently by sex, were not available in our study. Fourth, data on thrombus composition or pathological examination were not available in the RESCUE BT trial, preventing us from investigating whether sex modified the association between tirofiban and outcomes according to stroke etiology or thrombus composition. Therefore, large randomized controlled trials or cohort studies are warranted to validate our findings.

## Conclusion

In this secondary analysis of the RESCUE BT randomized trial, no significant interaction between sex and tirofiban administration was identified, and tirofiban was not associated with improved clinical outcomes compared to placebo in either sex. The lower odds of 90-day functional independence observed among women versus men in the tirofiban arm should be considered exploratory. These findings require validation in future adequately powered studies.

## Data Availability

The data analyzed in this study is subject to the following licenses/restrictions: the datasets generated during and/or analyzed during the current study are not publicly available but are available from the corresponding author on reasonable request. Requests to access these datasets should be directed to viperh1003@163.com.

## References

[1] Kain K Carter AM Bamford JM Grant PJ Catto AJ. Gender differences in coagulation and fibrinolysis in white subjects with acute ischemic stroke. J Thromb Haemost. (2003) 1:390–2. doi: 10.1046/j.1538-7836.2003.00040.x, 12871521

[2] Behl C. Oestrogen as a neuroprotective hormone. Nat Rev Neurosci. (2002) 3:433–42. doi: 10.1038/nrn846, 12042878

[3] Lisabeth LD Reeves MJ Baek J Skolarus LE Brown DL Zahuranec DB . Factors influencing sex differences in poststroke functional outcome. Stroke. (2015) 46:860–3. doi: 10.1161/STROKEAHA.114.007985, 25633999 PMC4342324

[4] Eriksson M Åsberg S Sunnerhagen KS von Euler M. Sex differences in stroke care and outcome 2005-2018: observations from the Swedish stroke register. Stroke. (2021) 52:3233–42. doi: 10.1161/STROKEAHA.120.033893, 34187179

[5] Renoux C Coulombe J Li L Ganesh A Silver L Rothwell PM. Confounding by pre-morbid functional status in studies of apparent sex differences in severity and outcome of stroke. Stroke. (2017) 48:2731–8. doi: 10.1161/STROKEAHA.117.018187, 28798261 PMC5610564

[6] Appelros P Stegmayr B Terént A. Sex differences in stroke epidemiology: a systematic review. Stroke. (2009) 40:1082–90. doi: 10.1161/STROKEAHA.108.540781 19211488

[7] Weber R Krogias C Eyding J Bartig D Meves SH Katsanos AH . Age and sex differences in ischemic stroke treatment in a Nationwide analysis of 1.11 million hospitalized cases. Stroke. (2019) 50:3494–502. doi: 10.1161/STROKEAHA.119.026723, 31623547

[8] Foerch C Misselwitz B Humpich M Steinmetz H Neumann-Haefelin T Sitzer M. Sex disparity in the access of elderly patients to acute stroke care. Stroke. (2007) 38:2123–6. doi: 10.1161/STROKEAHA.106.478495 17525398

[9] Reeves MJ Prager M Fang J Stamplecoski M Kapral MK. Impact of living alone on the care and outcomes of patients with acute stroke. Stroke. (2014) 45:3083–5. doi: 10.1161/STROKEAHA.114.006520, 25139877

[10] Tsai JP Nguyen TN Pujara DK Fifi JT Sundararajan S Schaafsma JD . Sex-based differences in endovascular thrombectomy outcomes for large ischemic stroke: a SELECT2 subanalysis. Stroke. (2025) 56:294–304. doi: 10.1161/STROKEAHA.124.049307 39744837

[11] Sun D Guo X Ling L Jiao L Nguyen TN Abdalkader M . Sex-related differences in endovascular treatment outcomes for acute Large infarcts: the ANGEL-ASPECT subanalysis. Stroke. (2025) 56:2033–42. doi: 10.1161/STROKEAHA.124.050025, 40340582

[12] Sun D Raynald N Huo X Jia B Tong X Ma G . Sex-related differences in outcomes of endovascular treatment for anterior circulation large vessel occlusion. Stroke. (2023) 54:327–36. doi: 10.1161/STROKEAHA.122.041195 36689588

[13] deIR Ridr Fransen PSS Beumer D Berkhemer OA Berg V Wermer MJ . Is intra-arterial treatment for acute ischemic stroke less effective in women than in men? Interv Neurol. (2016) 5:174–8. doi: 10.1159/000447331 PMC5075805 27781046

[14] Yang M Huo X Miao Z Wang Y. Platelet glycoprotein IIb/IIIa receptor inhibitor tirofiban in acute ischemic stroke. Drugs. (2019) 79:515–29. doi: 10.1007/s40265-019-01078-0, 30838514

[15] Zhao W Li S Li C Wu C Wang J Xing L . Effects of tirofiban on neurological deterioration in patients with acute ischemic stroke: a randomized clinical trial. JAMA Neurol. (2024) 81:594–602. doi: 10.1001/jamaneurol.2024.0868, 38648030 PMC11036313

[16] Tao C Liu T Cui T Liu J Li Z Ren Y . Early tirofiban infusion after intravenous thrombolysis for stroke. N Engl J Med. (2025) 393:1191–201. doi: 10.1056/NEJMoa2503678, 40616232

[17] Z W S J K W H J G C H W . Tirofiban for stroke without large or medium-sized vessel occlusion. N Engl J Med. (2023) 388:2025–203. doi: 10.1056/NEJMoa2214299 37256974

[18] Lin L Liu F Yi T Zhu Y Yang J Zhao Y . Tirofiban on first-pass recanalization in acute stroke endovascular thrombectomy: the OPTIMISTIC randomized clinical trial. JAMA Netw Open. (2025) 8:e255308. doi: 10.1001/jamanetworkopen.2025.5308, 40244586 PMC12006867

[19] RESCUE BT Trial Investigators Qiu Z Li F Sang H Luo W Liu S . Effect of intravenous tirofiban vs placebo before endovascular thrombectomy on functional outcomes in large vessel occlusion stroke: the RESCUE BT randomized clinical trial. JAMA. (2022) 328:543–53. doi: 10.1001/jama.2022.12584, 35943471 PMC9364124

[20] von Kummer R Broderick JP Campbell BCV Demchuk A Goyal M Hill MD . The Heidelberg bleeding classification: classification of bleeding events after ischemic stroke and reperfusion therapy. Stroke. (2015) 46:2981–6. doi: 10.1161/STROKEAHA.115.010049, 26330447

[21] Dula AN Mlynash M Zuck ND Albers GW Warach SJ DEFUSE 3 Investigators. Neuroimaging in ischemic stroke is different between men and women in the DEFUSE 3 cohort. Stroke. (2020) 51:481–8. doi: 10.1161/STROKEAHA.119.028205, 31826731 PMC7121881

[22] Yu AYX Vatanpour S Ganesh A Field TS Barber PA Choi PMC . Sex differences in outcomes after Tenecteplase for minor stroke: a subanalysis of the TEMPO-2 trial. J Am Heart Assoc. (2025) 14:e039154. doi: 10.1161/JAHA.124.039154, 40240937 PMC12184240

[23] McKevitt C Coshall C Tilling K Wolfe C. Are there inequalities in the provision of stroke care? Analysis of an inner-city stroke register. Stroke. (2005) 36:315–20. doi: 10.1161/01.STR.0000152332.32267.19, 15618440

[24] Qu HL Sun XY He C Chen HS. Sex differences in the dual antiplatelet therapy versus alteplase for patients with minor nondisabling acute ischemic stroke: a secondary analysis of the ARAMIS study. CNS Drugs. (2024) 38:649–59. doi: 10.1007/s40263-024-01096-x, 38806883

[25] Rong B Guo Z Gao L Yang Y Zi W Qiu Z . Association of tirofiban treatment with outcomes following endovascular therapy in cardioembolic stroke: insights from the RESCUE BT randomized trial. Eur J Med Res. (2023) 28:473. doi: 10.1186/s40001-023-01406-x, 37915101 PMC10621173

[26] Jolugbo P Ariëns RAS. Thrombus composition and efficacy of thrombolysis and thrombectomy in acute ischemic stroke. Stroke. (2021) 52:1131–42. doi: 10.1161/STROKEAHA.120.032810, 33563020 PMC7610448

[27] Hund HM Boodt N Hansen D Haffmans WA Lycklama À Nijeholt GJ Hofmeijer J . Association between thrombus composition and stroke etiology in the MR CLEAN registry biobank. Neuroradiology. (2023) 65:933–43. doi: 10.1007/s00234-023-03115-y, 36695859 PMC10105654

[28] Carcel C Harris K Peters SAE Sandset EC Balicki G Bushnell CD . Representation of women in stroke clinical trials: a review of 281 trials involving more than 500,000 participants. Neurology. (2021) 97:e1768–74. doi: 10.1212/WNL.0000000000012767, 34645708

[29] Carcel C Reeves M. Under-enrollment of women in stroke clinical trials: what are the causes and what should be done about it? Stroke. (2021) 52:452–7. doi: 10.1161/STROKEAHA.120.033227 33493049

