# Supplementary material for: Association between tirofiban administration and sex in outcomes following endovascular treatment for large vessel occlusion stroke
Source: Front Neurol. 2026 Sep 9;17:1850978. doi: 10.3389/fneur.2026.1850978 (PMC13597796; doi:10.3389/fneur.2026.1850978)
Supplement: Supplementary file 1 [file Table_1.DOCX]

**Supplementary Materials**

**Supplemental Table 1.** Baseline Characteristics Between Male and Female Among LAA Patients

**Supplemental Table 2.** Baseline Characteristics by Sex, Stratified by Treatment Arms Among LAA Patients

**Supplemental Table 3.** Outcomes by sex, stratified by treatment arms with age included as a covariate

**Supplemental Table 4.** Interaction Between Sex and Stroke Etiology for the Primary Outcome Within the Tirofiban Arm

**Supplemental Table 5.** Outcomes by Sex, Stratified by Treatment Arms Among LAA Patients

**Supplemental Table 6.** Baseline Characteristics by Treatment Arms, Stratified by Sex

**Supplemental Table 7.** Outcomes by Treatment Arms, Stratified by Sex

**Supplemental Figure 1.** Flowchart of Analysis Comparing Male Versus Female, Stratified by Treatment Arm

**Supplemental Figure 2.** Flowchart of Analysis Comparing Tirofiban Versus Placebo, Stratified by Sex

**Supplemental Figure 3.** Modified Rankin Scale Score Distribution at 90 Days Stratified by Sex Among LAA Patients

**Supplemental Figure 4.** Modified Rankin Scale Score Distribution at 90 Days by Treatment Arm, Stratified by Sex Among LAA Patients

**Supplemental Figure 5.** Association of Clinical Features With the Probability Of mRS Score 0–2 at 90 Days in the Tirofiban Arm

**Supplemental Figure 6.** Association Of Clinical Features With the Probability of Death At 90 Days in the Tirofiban Arm

**Supplemental Figure 7.** Association Of Clinical Features With the Probability of mRS Score 0–2 at 90 Days in the Placebo Arm

**Supplemental Figure 8.** Association Of Clinical Features With the Probability of Death at 90 Days in the Placebo Arm

**Supplemental Table 1.** Baseline Characteristics Between Male and Female Among LAA Patients

| **Baseline characteristics** | **Overall (n = 435)** | **Sex difference among LAA patients** | | ***P* value** |
| --- | --- | --- | --- | --- |
|  |  | **Female (n = 124)** | **Male (n = 311)** |  |
| Age, median (IQR) | 65 (56–72) | 71 (64–77) | 63 (54–70) | <0.001 |
| Hypertension, n (%) | 280 (64.4) | 89 (71.8) | 191 (61.4) | 0.054 |
| Diabetes, n (%) | 120 (27.6) | 46 (37.1) | 74 (23.8) | 0.007 |
| Hyperlipidemia, n (%) | 85 (19.5) | 27 (21.8) | 58 (18.6) | 0.543 |
| Past stroke, n (%) | 68 (15.6) | 22 (17.7) | 46 (14.8) | 0.536 |
| Atrial fibrillation, n (%) | 37 (8.5) | 25 (20.2) | 12 (3.9) | <0.001 |
| Smoking, n (%) | 152 (34.9) | 1 (0.8) | 151 (48.6) | <0.001 |
| SBP, mean ± SD | 149 ± 24 | 149 ± 25 | 150 ± 23 | 0.256 |
| DBP, median (IQR) | 85 (76–96) | 82 (72–93) | 86 (78–97) | 0.014 |
| Glucose, median (IQR) | 6.97 (5.70–9.30) | 7.58 (6.17–10.70) | 6.72 (5.60–8.72) | <0.001 |
| Prestroke mRS more than 1, n (%) | 8 (1.8) | 1 (0.8) | 7 (2.3) | 0.537 |
| Stroke characteristics |  |  |  |  |
| Baseline NIHSS score, median (IQR) | 14 (10–19) | 17 (13–20) | 13 (9–17) | <0.001 |
| Baseline ASPECTS, median (IQR) | 8 (7–9) | 8 (6.75–9) | 8 (7–9) | 0.835 |
| Occlusion site (%) |  |  |  | 0.131 |
| ICA | 70 (16.1) | 26 (21.0) | 44 (14.1) |  |
| MCA-M1 | 313 (72.0) | 81 (65.3) | 232 (74.6) |  |
| MCA-M2 | 52 (12.0) | 17 (13.7) | 35 (11.3) |  |
| Time metrics, median (IQR), min |  |  |  |  |
| Last known well to puncture | 480 (303–714) | 440 (278–672) | 493 (322–738) | 0.028 |
| Puncture to recanalization or procedure completion | 82 (50–132) | 81 (46.50–130) | 83 (51–132) | 0.366 |

Continuous variables are presented as mean ± SD or median (IQR), as appropriate. Categorical variables are presented as n (%).

Abbreviations: ASPECTS, Alberta Stroke Program Early CT Score; DBP, diastolic blood pressure; ICA, internal carotid artery; IQR, interquartile range; MCA, middle cerebral artery; NIHSS, National Institutes of Health Stroke Scale; SBP, systolic blood pressure.

**Supplemental Table 2.** Baseline Characteristics by Sex, Stratified by Treatment Arms Among LAA Patients

| **Baseline characteristics** | **Tirofiban (n = 197)** | | | **Placebo (n = 238)** | | |
| --- | --- | --- | --- | --- | --- | --- |
|  | **Female (n = 64)** | **Male (n = 133)** | ***P* value** | **Female (n = 60)** | **Male (n = 178)** | ***P* value** |
| Age, mean ± SD | 68 ± 10 | 61 ± 11 | <0.001 | 70 ± 10 | 62 ± 11 | <0.001 |
| Hypertension, n (%) | 45 (70.3) | 77 (57.9) | 0.127 | 44 (73.3) | 114 (64.0) | 0.246 |
| Diabetes, n (%) | 27 (42.2) | 27 (20.3) | 0.002 | 19 (31.7) | 47 (26.4) | 0.535 |
| Hyperlipidemia, n (%) | 16 (25.0) | 29 (21.8) | 0.75 | 11 (18.3) | 29 (16.3) | 0.868 |
| Past stroke, n (%) | 11 (17.2) | 16 (12.0) | 0.445 | 11 (18.3) | 30 (16.9) | 0.948 |
| Atrial fibrillation, n (%) | 13 (20.3) | 5 (3.8) | <0.001 | 12 (20.0) | 7 (3.9) | <0.001 |
| Smoking, n (%) | 0 (0.0) | 62 (46.6) | <0.001 | 1 (1.7) | 89 (50.0) | <0.001 |
| SBP, mean ± SD | 150 ± 24 | 150 ± 25 | 0.883 | 147 ± 27 | 150 ± 22 | 0.417 |
| DBP, median (IQR) | 83 (70–96) | 87 (72–101) | 0.058 | 83 (72–95) | 86 (78–99) | 0.12 |
| Glucose, median (IQR) | 7.69 (6.17–11.79) | 6.75 (5.45–8.05) | 0.001 | 7.37 (6.08–10.20) | 6.72 (5.63–9.41) | 0.115 |
| Prestroke mRS more than 1, n (%) | 1 (1.6) | 4 (3.0) | 0.904 | 0 (0.0) | 3 (1.7) | 0.732 |
| Stroke characteristics |  |  |  |  |  |  |
| Baseline NIHSS score, median (IQR) | 15 (11–19) | 14 (9–20) | 0.038 | 18 (14–20) | 13 (9–17) | <0.001 |
| Baseline ASPECTS, median (IQR) | 8 (7–9) | 8 (7–9) | 0.341 | 7 (6–9) | 8 (7–9) | 0.189 |
| Occlusion site (%) |  |  | 0.472 |  |  | 0.147 |
| ICA | 13 (20.3) | 18 (13.5) |  | 13 (21.7) | 26 (14.6) |  |
| MCA-M1 | 46 (71.9) | 104 (78.2) |  | 35 (58.3) | 128 (71.9) |  |
| MCA-M2 | 5 (7.8) | 11 (8.3) |  | 12 (20.0) | 24 (13.5) |  |
| Time metrics in minutes, median (IQR), min |  |  |  |  |  |  |
| Last known well to puncture | 460 (304–636) | 520 (310–725) | 0.182 | 435 (250–685) | 484 (336–745) | 0.079 |
| Puncture to recanalization or procedure completion | 82 (47–130) | 82 (50–125) | 0.83 | 74 (49–130) | 90 (54–133) | 0.314 |

Data are presented as median (interquartile range) for continuous variables and n (%) for categorical variables.

Abbreviations: ASPECTS, Alberta Stroke Program Early CT Score; DBP, diastolic blood pressure; ICA, internal carotid artery; IQR, interquartile range; MCA, middle cerebral artery; NIHSS, National Institutes of Health Stroke Scale; SBP, systolic blood pressure.

**Supplemental Table 3.** Outcomes by sex, stratified by treatment arms with age included as a covariate

| **Outcomes** | **Tirofiban (n = 463)** | | | | **Placebo (n = 485)** | | | | ***P* for interaction** |
| --- | --- | --- | --- | --- | --- | --- | --- | --- | --- |
|  | **Female**  **(n = 200)** | **Male**  **(n = 263)** | **aOR**  **(95% CI)**^a^ | ***P* value** | **Female**  **(n = 191)** | **Male**  **(n = 294)** | **aOR**  **(95% CI)**^a^ | ***P* value** |  |
| mRS 0–2 at 90 days, n (%)^b^ | 83 (41.5) | 145 (55.1) | 0.62 (0.39–0.99) | 0.045 | 81 (42.4) | 138 (46.9) | 1.07 (0.67–1.70) | 0.777 | 0.083 |
| mRS scores at 90 days, median (IQR) ^c^ | 3 (1-5) | 2 (1-4) | 0.81 (0.55–1.18) | 0.27 | 3 (1-5) | 3 (1-4) | 1.05 (0.72–1.53) | 0.799 | 0.385 |
| mRS 0–1 at 90 days, n (%)^b^ | 58 (29.0) | 110 (41.8) | 0.60 (0.37–0.98) | 0.039 | 55 (28.8) | 102 (34.7) | 0.93 (0.56–1.53) | 0.776 | 0.209 |
| mRS 0–3 at 90 days, n (%)^b^ | 113 (56.5) | 180 (68.4) | 0.76 (0.46–1.24) | 0.27 | 107 (56.0) | 192 (65.3) | 1.05 (0.66–1.69) | 0.827 | 0.296 |
| Mortality at 90 days, n (%)^b^ | 40 (20.0) | 44 (16.7) | 0.82 (0.46–1.48) | 0.514 | 36 (18.8) | 46 (15.6) | 0.81 (0.46–1.42) | 0.458 | 0.744 |
| sICH within 48 hours, n (%)^b^ | 20 (10.0) | 25 (9.5) | 0.74 (0.36–1.51) | 0.41 | 13 (6.8) | 18 (6.1) | 0.75 (0.31–1.82) | 0.530 | 0.879 |
| Any ICH, n (%)^b^ | 78 (39.0) | 83 (31.7) | 1.05 (0.67–1.65) | 0.83 | 60 (31.6) | 75 (25.6) | 0.97 (0.60–1.56) | 0.898 | 0.685 |

Data are presented as n (%) or median (interquartile range), as appropriate.

^a^ Adjusted for age, atrial fibrillation, baseline NIHSS score, baseline ASPECTS, last-seen-well to puncture time, occlusion site, smoking, and stroke etiology.

^b^ The odds ratios were estimated from binary logistic regression models.

^c^ The common odds ratio was estimated from ordinal logistic regression models and indicated the odds of improvement toward a lower mRS score. Men served as the reference group; therefore, a common odds ratio greater than 1 indicates more favorable functional outcomes in women.

^d^ *P* values for interaction were derived from the sex-by-treatment interaction term in the corresponding adjusted regression models.

Abbreviations: ASPECTS, Alberta Stroke Program Early Computed Tomography Score; CI, confidence interval; ICH, intracranial hemorrhage; IQR, interquartile range; mRS, modified Rankin Scale; NIHSS, National Institutes of Health Stroke Scale; OR, odds ratio; sICH, symptomatic intracranial hemorrhage.

**Supplemental Table 4.** Interaction Between Sex and Stroke Etiology for the Primary Outcome Within the Tirofiban Arm

| **Outcomes** | ***P* for interaction^a^** |
| --- | --- |
| mRS 0–2 at 90 days^b^ | 0.237 |
| mRS scores at 90 days^c^ | 0.446 |
| mRS 0–1 at 90 days^b^ | 0.451 |
| mRS 0–3 at 90 days^b^ | 0.446 |
| Mortality at 90 days^b^ | 0.821 |
| sICH within 48 hours^b^ | 0.479 |
| Any ICH^b^ | 0.473 |

^a^ *P* values for interaction were derived from the sex*stroke etiology interaction term in the corresponding adjusted regression models.

^b^ The odds ratios were estimated from binary logistic regression models.

^c^ The common odds ratio was estimated from ordinal logistic regression models

Abbreviations: ICH, intracranial hemorrhage; sICH, symptomatic intracranial hemorrhage; mRS, modified Rankin Scale.

**Supplemental Table 5.** Outcomes by Sex, Stratified by Treatment Arms Among LAA Patients

| **Outcomes** | **Tirofiban (n = 197)** | | | | | | **Placebo (n = 238)** | | | | | | ***P* for interaction^d^** |
| --- | --- | --- | --- | --- | --- | --- | --- | --- | --- | --- | --- | --- | --- |
|  | **Female (n = 64)** | **Male (n = 133)** | **OR**  **(95% CI)** | ***P* value** | **aOR**  **(95% CI)^a^** | ***P* value** | **Female**  **(n = 60)** | **Male (n = 178)** | **OR**  **(95% CI)** | ***P* value** | **aOR**  **(95% CI)^a^** | ***P* value** |  |
| Primary effectiveness outcome | | | | | | | | | | | | | |
| mRS 0–2 at 90 days, n (%)^b^ | 27 (42.2) | 71 (53.4) | 0.64 (0.35–1.16) | 0.14 | 0.64 (0.31–1.31) | 0.22 | 17 (28.3) | 76 (42.7) | 0.53 (0.28–1.00) | 0.05 | 1.20 (0.54–2.67) | 0.65 | 0.914 |
| Secondary effectiveness outcomes | | | | | | | | | | | | | |
| mRS score at 90 days, median (IQR)^c^ | 3 (1–4) | 2 (1–4) | 0.60 (0.36–1.02) | 0.06 | 0.69 (0.37–1.28) | 0.24 | 4 (2–6) | 3 (1–4) | 0.48 (0.28–0.81) | 0.01 | 1.00 (0.54–1.87) | 0.99 | 0.988 |
| mRS 0–1 at 90 days, n (%)^b^ | 17 (26.6) | 53 (39.8) | 0.55 (0.28–1.05) | 0.07 | 0.60 (0.27–1.32) | 0.21 | 11 (18.3) | 54 (30.3) | 0.52 (0.25–1.07) | 0.07 | 1.08 (0.44–2.63) | 0.86 | 0.673 |
| mRS 0–3 at 90 days, n (%)^b^ | 37 (57.8) | 94 (70.7) | 0.57 (0.31–1.06) | 0.07 | 0.68 (0.32–1.46) | 0.32 | 26 (43.3) | 114 (64.0) | 0.43 (0.24–0.78) | 0.01 | 0.89 (0.43–1.84) | 0.76 | 0.897 |
| Safety outcomes | | | | | | | | | | | | | |
| Mortality at 90 days, n (%)^b^ | 10 (15.6) | 18 (13.5) | 1.18 (0.51–2.73) | 0.69 | 0.80 (0.27–2.34) | 0.68 | 15 (25.0) | 29 (16.3) | 1.71 (0.84–3.47) | 0.14 | 0.82 (0.35–1.95) | 0.65 | 0.744 |
| sICH within 48 hours, n (%)^b^ | 3 (4.7) | 11 (8.3) | 0.54 (0.15–2.01) | 0.36 | 0.45 (0.10–1.92) | 0.28 | 7 (11.7) | 10 (5.6) | 2.21 (0.80–6.08) | 0.13 | 1.17 (0.34–4.01) | 0.80 | 0.121 |
| Any ICH, n (%)^b^ | 19 (29.7) | 38 (28.8) | 1.04 (0.54–2.01) | 0.90 | 0.96 (0.45–2.07) | 0.93 | 19 (31.7) | 36 (20.3) | 1.82 (0.94–3.50) | 0.07 | 1.25 (0.57–2.75) | 0.58 | 0.382 |

Data are presented as n (%) or median (interquartile range), as appropriate.

^a^ Adjusted for atrial fibrillation, baseline NIHSS score, baseline ASPECTS, last-seen-well to puncture time, occlusion site, and smoking.

^b^ The odds ratios were estimated from binary logistic regression models.

^c^ The common odds ratio was estimated from ordinal logistic regression models and indicated the odds of improvement toward a lower mRS score. Men served as the reference group; therefore, a common odds ratio greater than 1 indicates more favorable functional outcomes in women.

^d^ *P* values for interaction were derived from the sex-by-treatment interaction term in the corresponding adjusted regression models.

Abbreviations: ASPECTS, Alberta Stroke Program Early Computed Tomography Score; CI, confidence interval; ICH, intracranial hemorrhage; IQR, interquartile range; mRS, modified Rankin Scale; NIHSS, National Institutes of Health Stroke Scale; OR, odds ratio; sICH, symptomatic intracranial hemorrhage.

**Supplemental Table 6.** Baseline Characteristics by Treatment Arms, Stratified by Sex

| **Baseline characteristics** | **Female (n = 391)** | | | **Male (n = 557)** | | | |
| --- | --- | --- | --- | --- | --- | --- | --- |
|  | **Tirofiban**  **(n = 200)** | **Placebo**  **(n = 191)** | ***P* value** | **Tirofiban**  **(n = 263)** | **Placebo**  **(n = 294)** | | ***P* value** |
| Age, median (IQR) | 72 (62–77) | 71 (64–77) | 0.79 | 65 (55–72) | 64 (55–72) | | 0.63 |
| Hypertension, n (%) | 111 (55.5) | 110 (57.6) | 0.68 | 140 (53.2) | 163 (55.4) | | 0.60 |
| Diabetes, n (%) | 55 (27.5) | 43 (22.5) | 0.26 | 44 (16.7) | 62 (21.1) | | 0.19 |
| Hyperlipidemia, n (%) | 34 (17.0) | 23 (12.0) | 0.17 | 40 (15.2) | 35 (11.9) | | 0.25 |
| Past stroke, n (%) | 34 (17.0) | 35 (18.3) | 0.73 | 38 (14.4) | 54 (18.4) | | 0.21 |
| Atrial fibrillation, n (%) | 101 (50.5) | 81 (42.4) | 0.11 | 65 (24.7) | 66 (22.4) | | 0.53 |
| Smoking, n (%) | 0 (0) | 2 (1.0) | 0.15 | 100 (38.0) | 120 (40.8) | | 0.50 |
| SBP, median (IQR), mmHg | 145 (126–164) | 141 (126–158) | 0.2 | 145 (130–160) | 146 (131–160) | | 0.83 |
| DBP, median (IQR), mmHg | 83 (75–94) | 81 (73–92) | 0.52 | 84 (76–93) | 85 (77–98) | | 0.13 |
| Glucose, median (IQR), mmol/l | 7.4 (6.2–9.7) | 7.1 (5.8–8.8) | 0.09 | 6.7 (5.4–7.8) | 6.8 (5.7–8.7) | | 0.06 |
| Prestroke mRS, n (%) | | | 0.32 |  | | | 0.30 |
| 0-1 | 196 (98.0) | 184 (96.3) |  | 255 (97.0) | 289 (98.3) | |  |
| ≥2 | 4 (2) | 7 (3.7) |  | 8 (3.0) | 5 (1.7) | |  |
| Stroke characteristics | | | | | | | |
| Baseline NIHSS, median (IQR) | 16 (12–20) | 17 (14–20) | 0.13 | 15 (11–18) | 15 (10–19) | | 0.87 |
| Baseline ASPECTS, median (IQR) | 8 (7–9) | 7 (6–9) | 0.06 | 8 (7–9) | 8 (7–9) | | 0.72 |
| Stroke etiology, n (%) | | | 0.51 |  | | 0.06 | |
| Large artery atherosclerosis | 64 (32.0) | 60 (31.4) |  | 133 (50.6) | 178 (60.5) | |  |
| Cardioembolism | 119 (59.5) | 108 (56.5) |  | 93 (35.4) | 86 (29.3) | |  |
| Other or Unknown | 17 (8.5) | 23 (12.0) |  | 37 (14.1) | 30 (10.2) | |  |
| Occlusion site, n (%) | | | 0.45 |  | | | 0.90 |
| ICA | 47 (23.5) | 46 (24.1) |  | 49 (18.6) | 52 (17.7) | |  |
| MCA-M1 | 128 (64.0) | 113 (59.2) |  | 177 (67.3) | 197 (67.0) | |  |
| MCA-M2 | 25 (12.5) | 32 (16.8) |  | 37 (14.1) | 45 (15.3) | |  |
| Time metrics in minutes, median (IQR), min | | | | | | | |
| Last known well to puncture | 366 (263–568) | 325 (205–565) | 0.08 | 435 (280–680) | 440 (270–662) | | 0.99 |
| Puncture to recanalization or procedure completion | 65 (39–98) | 60 (39–100) | 0.92 | 68 (42–108) | 75 (45–121) | | 0.11 |

Data are presented as median (interquartile range) for continuous variables and n (%) for categorical variables.

Abbreviations: ASPECTS, Alberta Stroke Program Early CT Score; DBP, diastolic blood pressure; ICA, internal carotid artery; IQR, interquartile range; MCA, middle cerebral artery; NIHSS, National Institutes of Health Stroke Scale; SBP, systolic blood pressure.

**Supplemental Table 7.** Outcomes by Treatment Arms, Stratified by Sex

| **Outcomes** | **Female (n = 391)** | | | | | | **Male (n = 557)** | | | | | | ***P* for interaction^d^** |
| --- | --- | --- | --- | --- | --- | --- | --- | --- | --- | --- | --- | --- | --- |
|  | **Tirofiban**  **(n = 200)** | **Placebo**  **(n = 191)** | **OR**  **(95% CI)** | ***P* value** | **aOR**  **(95% CI)^a^** | ***P* value** | **Tirofiban**  **(n = 26 3)** | **Placebo**  **(n = 294)** | **OR**  **(95% CI)** | ***P* value** | **aOR**  **(95% CI)^a^** | ***P* value** |  |
| Primary effectiveness outcome | | | | | | | | | | | | | |
| mRS 0–2 at 90 days, n (%)^b^ | 83 (41.5) | 81 (42.4) | 0.96 (0.65–1.44) | 0.86 | 0.92 (0.60–1.41) | 0.70 | 145 (55.1) | 138 (46.9) | 1.39 (0.995–1.94) | 0.054 | 1.43 (0.99–2.06) | 0.06 | 0.103 |
| Secondary effectiveness outcomes | | | | | | | | | | | | | |
| mRS score at 90 days, median (IQR)^c^ | 3 (1–5) | 3 (1–5) | 1.05 (0.74–1.48) | 0.81 | 1.00 (0.70–1.43) | 1.00 | 2 (1–4) | 3 (1–4) | 1.18 (0.88–1.59) | 0.26 | 1.14 (0.85–1.53) | 0.39 | 0.465 |
| mRS 0–1 at 90 days, n (%)^b^ | 58 (29.0) | 55 (28.8) | 1.01 (0.65–1.56) | 0.96 | 0.95 (0.60–1.51) | 0.83 | 110 (41.8) | 102 (34.7) | 1.35 (0.96–1.91) | 0.08 | 1.36 (0.93–1.98) | 0.11 | 0.219 |
| mRS 0–3 at 90 days, n (%)^b^ | 113 (56.5) | 107 (56.0) | 1.02 (0.68–1.52) | 0.92 | 0.93 (0.61–1.42) | 0.74 | 180 (68.4) | 192 (65.3) | 1.15 (0.81–1.64) | 0.43 | 1.20 (0.82–1.77) | 0.35 | 0.403 |
| Safety outcomes | | | | | | | | | | | | | |
| Mortality at 90 days, n (%)^b^ | 40 (20.0) | 36 (18.8) | 1.08 (0.65–1.78) | 0.77 | 1.11 (0.66–1.87) | 0.68 | 44 (16.7) | 46 (15.6) | 1.08 (0.69–1.70) | 0.73 | 1.05 (0.65–1.71) | 0.84 | 0.811 |
| sICH within 48 hours, n (%)^b^ | 20 (10.0) | 13 (6.8) | 1.51 (0.73–3.13) | 0.27 | 1.44 (0.68–3.08) | 0.34 | 25 (9.5) | 18 (6.1) | 1.61 (0.86–3.03) | 0.14 | 1.45 (0.75–2.82) | 0.27 | 0.923 |
| Any ICH, n (%)^b^ | 78 (39.0) | 60 (31.6) | 1.39 (0.91–2.10) | 0.13 | 1.36 (0.88–2.09) | 0.17 | 83 (31.7) | 75 (25.6) | 1.35 (0.93–1.95) | 0.11 | 1.28 (0.87–1.88) | 0.21 | 0.696 |

Data are presented as n (%) or median (interquartile range), as appropriate.

^a^ Adjusted for atrial fibrillation, baseline NIHSS score, baseline ASPECTS, last-seen-well to Puncture time, occlusion site, smoking, and stroke etiology.

^b^ The odds ratios were estimated from binary logistic regression models.

^c^ The common odds ratio was estimated from ordinal logistic regression models and indicated the odds of improvement of 1 point on the mRS, with a common odds ratio greater than 1 favoring the tirofiban treatment.

^d^ P values for interaction were derived from the sex-by-treatment interaction term in the corresponding adjusted regression models.

Abbreviations: ASPECTS, Alberta Stroke Program Early Computed Tomography Score; CI, confidence interval; ICH, intracranial hemorrhage; IQR, interquartile range; mRS, modified Rankin Scale; NIHSS, National Institutes of Health Stroke Scale; OR, odds ratio; sICH, symptomatic intracranial hemorrhage.

**Supplemental Figure 1.** Flowchart of Analysis Comparing Male Versus Female, Stratified by Treatment Arm

**Supplemental Figure 2.** Flowchart of Analysis Comparing Tirofiban Versus Placebo, Stratified by Sex

**Supplemental Figure 3.** Modified Rankin Scale Score Distribution at 90 Days Stratified by Sex Among LAA Patients

**Supplemental Figure 4.** Modified Rankin Scale Score Distribution at 90 Days by Treatment Arm, Stratified by Sex Among LAA Patients

**Supplemental Figure 5.** Association of Clinical Features With the Probability Of mRS Score 0–2 at 90 Days in the Tirofiban Arm

The probabilities of achieving an mRS score of 0–2 based on age, ASPECTS, baseline NIHSS score and puncture to recanalization time among patients in tirofiban arm are presented in A, B, C and D respectively. Solid lines indicate estimated probabilities of outcomes; shaded areas represent 95% CIs.

Abbreviations: ASPECTS, Alberta Stroke Program Early Computed Tomography Score; mRS, modified Rankin Scale; NIHSS, National Institutes of Health Stroke Scale; PTR, puncture to recanalization time.

**Supplemental Figure 6.** Association Of Clinical Features With the Probability of Death At 90 Days in the Tirofiban Arm

The probabilities of mortality based on age, ASPECTS, baseline NIHSS score and puncture to recanalization time among patients in tirofiban arm are presented in A, B, C and D respectively. Solid lines indicate estimated probabilities of outcomes; shaded areas represent 95% CIs.

Abbreviations are as in Table S3.

**Supplemental Figure 7.** Association Of Clinical Features With the Probability of mRS Score 0–2 at 90 Days in the Placebo Arm

The probabilities of achieving an mRS score of 0–2 based on age, ASPECTS, baseline NIHSS score and puncture to recanalization time among patients in placebo arm are presented in A, B, C and D respectively. Solid lines indicate estimated probabilities of outcomes; shaded areas represent 95% CIs.

Abbreviations are as in Table S3.

**Supplemental Figure 8.** Association Of Clinical Features With the Probability of Death at 90 Days in the Placebo Arm

The probabilities of mortality based on age, ASPECTS, baseline NIHSS score and puncture to recanalization time among patients in placebo arm are presented in A, B, C and D respectively. Solid lines indicate estimated probabilities of outcomes; shaded areas represent 95% CIs.

Abbreviations are as in Table S3.
